# Melatonin mitigates cadmium-induced oxidative damage and modulates polysaccharide biosynthesis in *Bletilla striata*

**DOI:** 10.3389/fpls.2025.1713721

**Published:** 2025-11-26

**Authors:** Deqiong Niu, Zhonghai Dao, Zhifeng Xiao, Ping Xiang, Shiyong Zhou, Jinrun Dong

**Affiliations:** Biochemical laboratory, College of Biological Science and Food Engineering, Southwest Forestry University, Kunming, China

**Keywords:** *Bletilla striata*, Cd stress, melatonin, oxidative damage polysaccharide biosynthesis, transcriptomic, metabolomic

## Abstract

**Introduction:**

Cadmium (Cd), a highly toxic heavy metal, threatens ecosystems and human health. *Bletilla striata* (*B*. *striata*), a medicinal plant valued for its polysaccharide-rich rhizomes, exhibits significant tolerance to Cd stress. Melatonin (MT), a phytohormone, has emerged as a key regulator of plant resilience to heavy metal toxicity. However, the mechanisms underlying MT-mediated biosynthesis of *B*. *striata* polysaccharides (BSP) under Cd exposure, as well as its role in alleviating Cd-induced oxidative damage, remain poorly characterized.

**Methods:**

Soil was passivated with 250 μmol/L CdCl_2_**·**2.5H_2_O for one month before transplanting one-year-old *B*. *striata* seedlings. One week after transplantation, the leaves of *B*. *striata* were sprayed with MT (50, 100, and 200 μmol/L) weekly. After 60 days, samples were collected and analyzed for physiological and biochemical indices (chlorophyll, osmoregulatory substances, Cd, reactive oxygen species, antioxidant enzyme activities, polysaccharides, flavonoids and saponins). Additionally, samples from CK, Cd-only, and Cd+50MT groups were collected for integrated transcriptomic and metabolomic analyses, and the transcriptome data were validated via qRT-PCR.

**Results:**

Our findings revealed that Cd stress substantially suppressed seedling growth, manifesting as significant reductions in fresh weight, dry weight, chlorophyll content, and proline accumulation relative to control plants. Under Cd stress, application of 50 μmol/L MT achieved the maximal reduction in the contents of hydrogen peroxide (H_2_O_2_) and malondialdehyde (MDA), while significantly enhancing the activities of antioxidant enzyme. Additionally, under the treatment of 50 μmol/L MT (Cd+50M), the contents of polysaccharides and flavonoids were also significantly increased. Multi-omics integration identified 21 differentially expressed genes (DEGs) and 4 differentially expressed metabolites (DEMs) prominently associated with the BSP biosynthetic pathway, among which 10 DEGs showed strong correlations with DEMs. Coexpression network analysis underscored MT-mediated modulation of pivotal BSP synthesis genes (e.g., *FRK1*, *FRK3*, *FRK6*, and *HK2*), with *MYB*, *MYB-related*, *bHLH*, and *bZIP* transcription factors implicated as central regulators. qRT-PCR analysis confirmed the reliability of the transcriptome data.

**Discussion:**

This study is helpful for understanding the regulatory network of MT in BSP biosynthesis under Cd stress and the role of MT in alleviating Cd-induced oxidative damage, thereby providing potential strategies for cultivating medicinal plants in heavy metal-contaminated environments.

## 1 Introduction

With accelerating urban industrialization, soil heavy metal pollution has become increasingly severe and is now recognized as a critical global environmental issue (Qiu et al., 2020). Among heavy metals, cadmium (Cd) ranks as one of the most hazardous substances and has been classified as a priority environmental pollutant (Cai et al., 2020; Yan et al., 2022). A 2022 study revealed that the geometric mean concentration of Cd in China’s agricultural soils reached 0.473 mg/kg, exceeding the farmland risk screening value by 58% (Wang et al., 2023). Statistical data indicate that 17.39% of cultivated land in China’s five major grain-producing regions exceeds the cadmium content standard, with this proportion showing a continuous increase. This has severely affected the physiological growth of crops, leading to declines in both yield and quality (Sang et al., 2018; Li et al., 2021). Studies have demonstrated that Cd stress disrupts redox balance, leading to the accumulation of reactive oxygen species (ROS) that triggers oxidative stress, disrupts cellular homeostasis, inhibits cellular processes, causes DNA damage and protein oxidation (Chai et al., 2022).

*Bletilla striata* (*B*. *striata*), a traditional Chinese medicinal herb, holds significant economic value and has garnered considerable attention due to its diverse pharmacological effects, including hemostasis, gastric mucosa protection, wound healing promotion, antibacterial activity, and anticancer properties (Tao et al., 2013). However, Chinese herbal medicines, including *B. striata*, possess a relatively limited market presence within the global traditional medicine industry, largely attributed to exogenous pollutant residues, such as heavy metals. These residues have not only compromised the quality and efficacy of medicinal plants but also acted as a key factor restricting the export trade of herbal products (Chen et al., 2021). It has been reported that high concentrations of Cd in roots disrupt redox homeostasis and elevate ROS levels, leading to poor root growth. For instance, under long-term exposure to high Cd (≥7.39 mg/kg), the biomass of *B. striata* significantly decreases (Yang et al., 2021). Additionally, Xu et al. also indicated that high Cd pollution remarkably reduces both the biomass of *B. striata* and the content of its polysaccharides (Xu et al., 2022). Among the diverse phytochemicals, BSP stand out as crucial characteristic compounds, and their content serves as a key indicator for evaluating the quality of *B. striata*. Cd exerts a dual effect on plant polysaccharide synthesis: it not only inhibits photosynthesis and reduces the availability of sugar precursors, thereby diminishing overall polysaccharide biosynthetic capacity, but also induces plants to actively modify the composition and structure of cell wall polysaccharides to sequester Cd in the apoplast, mitigating its cytotoxic effects. Yu et al. demonstrated that Cd downregulates the expression of key genes encoding Calvin cycle enzymes (e.g., *RbcS*, *PGK*, *GAPDH*) and disrupts the redox state of photosystem I (PSI), impairing electron transport. This results in reduced photosynthetic carbon fixation and a diminished supply of carbon skeletons necessary for the synthesis of nucleotide sugars (e.g., UDP-Glc, UDP-GalA), ultimately compromising the metabolic capacity for polysaccharide production at the source (Yu et al., 2025). In *Solanum lycopersicum* (tomato), Cd stress increased the contents of pectin, hemicellulose, and cellulose in roots by 3.41-fold, 2.79-fold, and 1.11-fold, respectively. This enhancement of cell wall Cd sequestration trapped 42-71% of Cd^2+^ outside the cells (Wang M. et al., 2025). Therefore, identifying effective exogenous agents to enhance the adaptability of *B. striata* to Cd stress has become a crucial strategy for its cultivation, yeild and medicinal quality.

Melatonin (MT), an indoleamine small molecule, functions as both a powerful antioxidant and an essential signaling modulator, thereby establishing its role as a “master regulator” in plants (Sun et al., 2021). In plants, MT is recognized as a broad-spectrum antioxidant that rapidly scavenges ROS and reactive nitrogen species (RNS) under stress conditions (Reiter et al., 2015). In addition, Exogenous MT has been shown to play an important role in alleviating the toxic effects of heavy metals on common plants and can reduce the accumulation of Cd in plant tissues (Tan et al., 2007; Wang et al., 2024). As a key signaling molecule for plants to cope with stress, MT can also bidirectionally regulate plant polysaccharide biosynthesis by reshaping the composition of cell wall polysaccharides, modulating carbon metabolic flux distribution, and mediating signaling pathways. Studies showed MT can achieve heavy metal sequestration and toxicity alleviation by promoting or inhibiting polysaccharide synthesis (Yang et al., 2023; Zeng et al., 2023). In addition, MT can also enhance osmotic protection by degrading storage polysaccharides and regulating structural polysaccharides (Liang et al., 2019). Moreover, under normal physiological conditions, exogenous MT application can provide sufficient carbon skeletons for polysaccharide synthesis by regulating the Calvin cycle and carbon metabolic flux distribution, while simultaneously modulating polysaccharide synthesis pathways to maintain the homeostasis of polysaccharide biosynthesis (Song et al., 2025). However, the regulatory mechanism of MT on polysaccharide biosynthesis of *B. striata* under Cd stress has not yet been reported.

In this study, we first investigated the effects of different concentrations of MT on the physiological and biochemical indices of *B. striata* seedlings under 250 μmol/L Cd stress, including chlorophyll, fresh weight, dry weight, osmotic adjustment substances, Cd concentration, ROS, antioxidant enzymes, polysaccharides, total flavonoids, and saponins. Then combined the transcriptomics and metabolomics analysis to elucidate the regulatory network of MT on BSP biosynthesis under Cd stress. This study will provide novel insights into the effects of MT on the accumulation of BSP under Cd stress and lay the foundation for unraveling the molecular mechanisms of MT in regulating the growth and quality of *B. striata* under Cd stress.

## 2 Materials and methods

### 2.1 Plant materials and treatments

*B. striata*, was obtained from Songming, Yunnan Province. The 1-year seedlings with consistent growth were used for the treatment. After one month of soil passivation treatment with 250 μmol/L CdCl_2_·2.5H_2_O, 1-year seedlings at uniform growth were transplanted in plastic pots (40 cm length × 20 cm width) with appropriate culture soil in a greenhouse, and the Cd concentration was screened by our laboratory. Different concentrations of MT were added after transplanting for one week. MT treatment was conducted by foliar application until the leaves were dripping. Spraying was done between 17:00-18:00 once every week. Control plants were grown in the same nutrient soil without Cd. Five treatments were designed in the experiment, that is CK (Control); Cd (250 μmol/L), Cd+50M (250 μmol/L Cd+50 μmol/L MT), Cd+100M (250 μmol/L Cd+100 μmol/L MT), Cd+200M (250 μmol/L Cd+200 μmol/L MT), and each treatment was repeated 3 times, 9 plants per pot. After 60 days of transplanting, the plants were collected, and the levels of chlorophyll, osmotic regulators, ROS, antioxidant enzymes, polysaccharides, total flavonoids and saponins were measured. Tuber samples from the CK, Cd, Cd+50M groups (three replicates for each) were collected and processed to transcriptomic and metabolomic analysis.

### 2.2 Determination of the chlorophyll, proline and soluble sugar content

In the detection of chlorophyll content, 0.1 g of *B. striata* leaf tissue was soaked in 80% acetone and then placed in darkness for 48 h. The absorbance of the chlorophyll extract was then measured at wavelengths of 470 nm, 645 nm, and 663 nm using a UV-visible spectrophotometer (Porra et al., 1989). For proline extraction, 0.5 g tuber samples were homogenized with 5 mL 3% sulfosalicylic acid solution and subjected to grinding with a precooled mortar and pestle. Then, the samples were boiling water bathed for 10 min and centrifuged at 12000 × g for 10 min at 4 °C, and the supernatant was collected for determination by ninhydrin colorimetric method with a UV-visible spectrophotometer (UV-5800, Shanghai Yuanxi Instrument Co. Ltd., China) at a wavelength of 520 nm (Li et al., 2022). For the soluble sugar content, 0.1 g of tuber tissue was homogenized in 1 mL of distilled water, extracted in a boiling water bath for 10 min, and centrifuged at 8000 r/min for 10 min. The supernatant was then determined using anthrone colorimetry with a UV-visible spectrophotometer at a wavelength of 620 nm (Zhang et al., 2023).

### 2.3 Determination of O_2_^-^, H_2_O_2_, and MDA content

The contents of superoxide anion (O_2_^-^), H_2_O_2_, and MDA in tubers were determined using Solarbio Assay Kits following the manufacturer’s instructions (Solarbio, Beijing, China). Per 0.4 g sample was ground to power using liquid nitrogen. The contents were extracted with a buffer according to the standard instructions O_2_^-^, H_2_O_2_ and MDA (BC1290, BC3595, BC0025) kits. Non-enzymatic antioxidants were noted at 530, 415, 532 and 600 nm wavelength with a UV-visible spectrophotometer (UV-5800, Shanghai Yuanxi Instrument Co. Ltd., China).

### 2.4 Determination of antioxidant enzyme activity

The activities of antioxidant enzymes including superoxide dismutase (SOD), peroxidase (POD), catalase (CAT), and ascorbate peroxidase (APX), in tubers were determined by spectrophotometry. Fresh tissue (0.5 g) was ground uniformly on ice with 2 mL of sodium phosphate buffer (pH 7.8). The homogenate was centrifuged at a speed of 10000 r/min at 4 °C for 20 min and the supernatant was used for the enzyme assays. The activities of antioxidant enzymes in tubers were determined using Solarbio Assay Kits following the manufacturer’s instructions (Solarbio, Beijing, China). The activity of SOD (BC5165) was measured by nitrotetraazolium blue sodium chloride (NBT) method, POD activity (BC0090) was evaluated by guaiacol method, and CAT activity (BC0205) was measured by ultraviolet absorption method. The activity of APX (BC0225) was also measured by ultraviolet absorption method.

### 2.5 Total polysaccharides, flavonoids and saponins content

Total polysaccharides were extracted by water extraction method described by Tang et al (Tang et al., 2019), and the protein was removed by Sevage method, and then the BSP content was subsequently determined by phenol-sulfur acid method. For the flavonoid content, 0.25 g of tuber powder was collected and sonicated with 70% ethanol at a solid-liquid ratio of 1:40 g/mL for 54 minutes. Then, the samples were centrifuged at 4000 rpm for 30 minutes, and 5 mL of the supernatant was collected in a 10 mL test tube with 0.4 mL of 5% NaNO_2_ solution and 0.4 mL of 10% Al(NO_3_)_3_ solution. Following a 7-minute equilibration at room temperature, 1 mL of 5% NaOH solution was added to the mixture, which was then transferred to a 10 mL volumetric flask. After 18 minutes, the concentration of the resulting solution was measured using a UV-visible spectrophotometer at a wavelength of 510 nm (Li et al., 2023). For saponins analysis, 10 mL 70% ethanol solution was added to 0.1 g of tubers samples, and the ultrasonic extraction of saponins was conducted for 0.9 h. After centrifuged at 4000 r/min for 30 min, 1 mL of the supernatant was removed and diluted fivefold. The content of saponins was determined with UV-visible spectrophotometry at 546 nm wavelength (Le Bot et al., 2022).

### 2.6 Determination of Cd concentration

For the determination of Cd content, tubers were individually collected, dried at 75°C to constant weight, and ground into a fine powder that was sieved through a 100-mesh sieve. A 0.2 g sample was accurately weighed into a 50 mL flat-bottom digestion tube, soaked overnight in 10 mL of 1:1 nitric acid solution, and subsequently digested in a graphite digestion furnace at 105°C until reduced to 2 mL. After cooling, 2 mL of 30% H_2_O_2_ solution was added to promote further digestion, which was continued until the volume reached approximately 1 mL. Upon cooling, the digest was transferred to a 25 mL volumetric flask, diluted to the mark with 0.1 mol/L nitric acid, filtered through a 0.45 μm aqueous membrane filter, and analyzed for Cd content using Inductively Coupled Plasma-Mass Spectrometry (ICP-MS) (Anjum et al., 2014).

### 2.7 Transcriptomic sequencing and analysis

Total RNA was extracted from different treatment groups of *B. striata* tubers using TRIzol reagent (Invitrogen, Carlsbad, CA, USA). The concentration and integrity of RNA samples were quantified using Qubit fluorescence quantitative analysis and automated electrophoresis systems (5300/5400 Fragment Analyzer). The cDNA library construction and sequencing were conducted by Hangzhou Lianchuan Biomedical Technology Co., Ltd. (www.lc-bio.com) using the Illumina high-throughput sequencing platform. The transcriptome was assembled *de novo* using Trinity 2.4.0 software (Grabherr et al., 2011). Transcript per million mapped reads (TPM) was calculated using Salmon software (Patro et al., 2017) to analyze the expression levels of individual genes (Unigenes) (Mortazavi et al., 2008). Differentially expressed Unigenes were screened using the R package edgeR, with the screening criteria being: log_2_ (fold change) >1 or log_2_ (fold change) <-1, and statistical significance (p-value < 0.05). In addition, the DIAMOND software was used for functional annotation of Unigenes, and the annotation was performed using six authoritative databases (NCBI_NR, GO, KEGG, Pfam, SwissProt, and eggNOG). GO and KEGG enrichment analyses were carried out by OmicStudio cloud platform (www.omicstudio.cn), respectively. All the pathway enrichment analysis were performed based on the KEGG database.

### 2.8 Targeted glucose metabolism analysis

The sugar contents were detected using MetWare Biotechnology Co., Ltd (http://www.metware.cn/) based on the gas chromatography-tandem mass spectrometry (GC-MS). After freeze-drying, the samples were ground using a mixer mill (30 Hz, 1.5 min). A 20 mg aliquot of the powder was weighed, and extraction solution (methanol:isopropanol:water = 3:3:2, v/v/v) was added. The mixture was vortexed, sonicated, and centrifuged; the supernatant was collected, mixed with an internal standard, evaporated to dryness under nitrogen gas, and derivatized with methoxyamine hydrochloride in pyridine and BSTFA. After filtration through a membrane, the samples were subjected to instrumental analysis using a DB-5MS column with helium as the carrier gas, and the selective ion monitoring (SIM) mode was employed. Metabolite content data were processed by unit variance scaling (UV), and the clustering heat map was drawn using R software. The metabolites significantly regulated between the two groups were determined by fold change (FC) ≥ 2 or ≤ 0.5.

### 2.9 RT-qPCR validation

Referring to the research method of Jiang et al (Jiang et al., 2024), the transcriptome data were verified by qRT−PCR. The 18S rRNA gene was used as the reference gene. Data analysis was performed using the 2^−ΔΔCT^ method (Zhao et al., 2024), which accurately quantified gene expression by comparing the expression of the target gene with that of the reference gene. For each experiment, three biological replicates were performed.

### 2.10 Statistical analysis

The data were organized using Microsoft Excel 2010. One-way analysis of variance (ANOVA) was performed on the physiological and biochemical indices using SPSS Statistics 20, with results expressed as mean ± standard error. Data visualization was conducted using Origin 2021. Significant differences the p-values < 0.05 level across various treatments are indicated by using distinct lowercase letters. Three biological replicates were set up for each treatment.

## 3 Results

### 3.1 Effects of MT on fresh weight, dry weight, chlorophyll and osmotic regulators of *B. striata*

**Figure 1:**
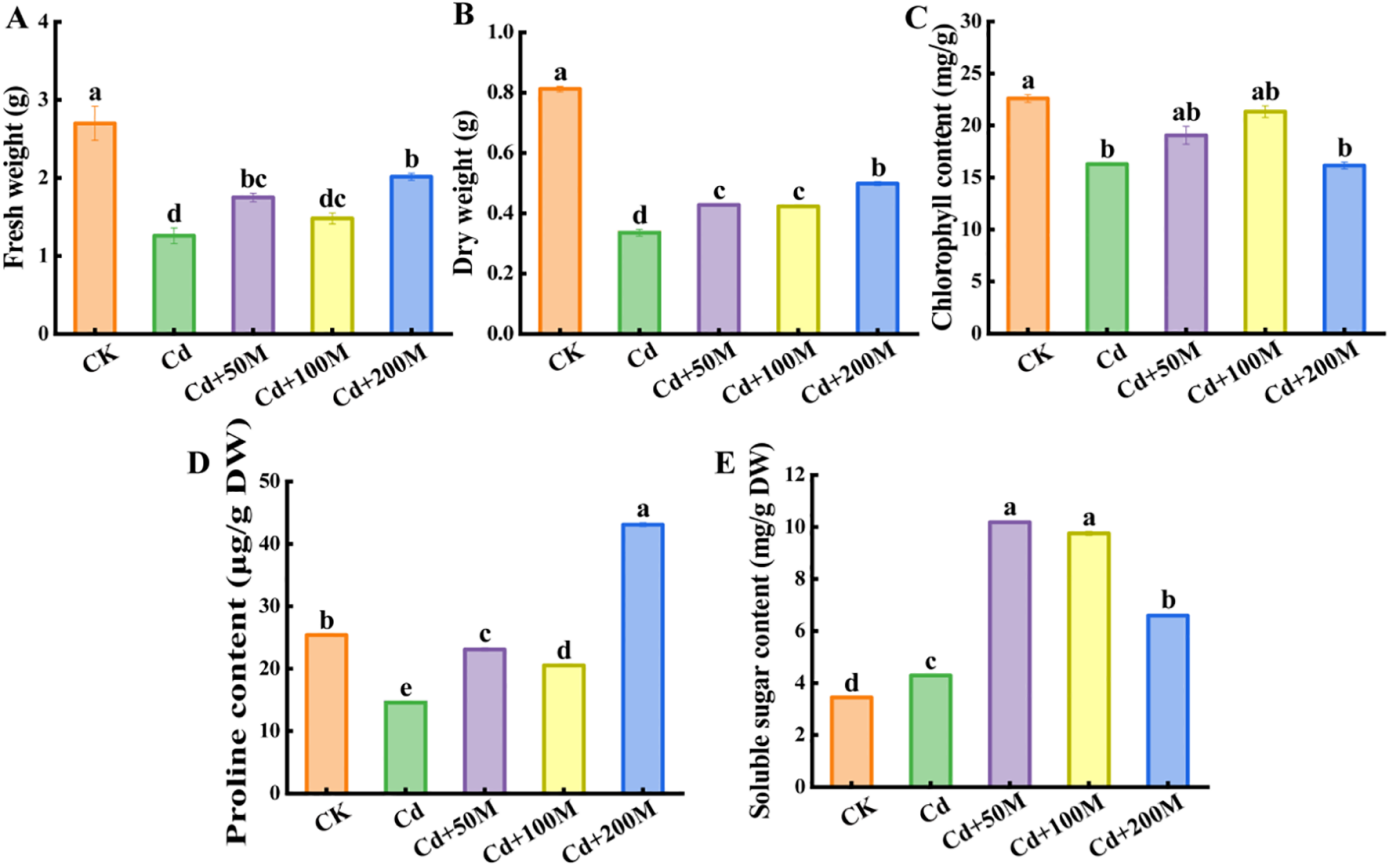
Effects of different MT treatments on the contents of fresh weight **(A)**, dry weight **(B)**, chlorophyll content **(C)**, proline content **(D)**, and soluble sugar content **(E)** of *B. striata* tubers under Cd stress. Letters (a, b, c, d, e) mean significant difference at p < 0.05.

To study the effect of MT treatment on the growth of *B. striata* under Cd stress, the phenotype of *B. striata* seedlings and accumulation of fresh weight, dry weight, chlorophyll, proline and soluble sugar of the tubers was measured. Compared with the control (CK), 250 μmol/L Cd significantly inhibited the growth of *B. striata* tubers. However, the addition of MT (50, 100 and 200 μmol/L) remarkable alleviated Cd toxicity (**Supplementary Figure S1**). As shown in **Figure 1**, compared with the CK, 250 μmol/L Cd significantly inhibited the growth of *B. striata*, and its fresh weight, dry weight, chlorophyll and proline content were significantly lower than that of the control. Furthermore, the fresh and dry weights of *B. striata* treated with different concentrations of MT under 250 μmol/L Cd stress were significantly lower than those of the CK. Compared with the Cd treatment alone (Cd), the fresh weight, dry weight, proline, and soluble sugar content increased significantly after adding 50 μmol/L MT (Cd+50M). After adding 100 μmol/L MT (Cd+100M), the chlorophyll, proline, and soluble sugar levels increased significantly, the fresh and dry weights content also increased, but the difference was not obvious. Except for chlorophyll content, the 200 μmol/L MT (Cd+200M) treatment demonstrated significant improvements in all four parameters. The results preliminarily indicated that MT application can promote the growth of *B. striata*.

### 3.2 Effects of MT on ROS and antioxidant enzyme activity of *B. striata*

**Figure 2:**
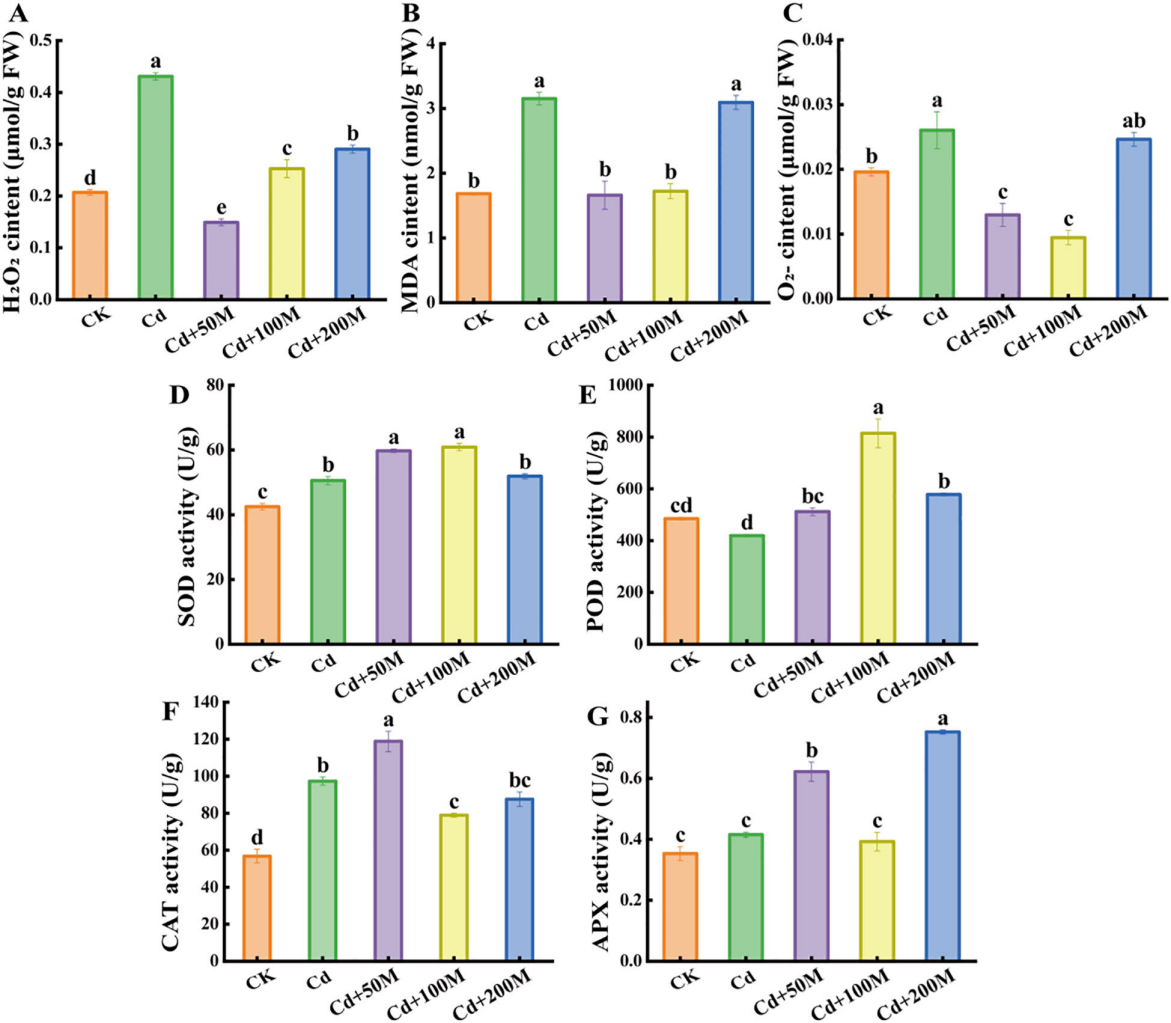
Effects of different MT treatments on the contents of H_2_O_2_**(A)**, MDA **(B)**, O_2_^-^**(C)**, SOD **(D)**, POD **(E)**, CAT **(F)** and APX **(G)** in *B. striata* tubers under Cd stress. Letters (a, b, c, d, e) mean significant difference at p < 0.05.

Cd-induced oxidative stress can lead to excessive accumulation of ROS and resulting macromolecular oxidative damage (Ali et al., 2020). The contents of H_2_O_2_, MDA and O_2_^-^ in *B. striata* tubers treated with 250 μmol/L Cd were significantly higher than that in the control (CK). Compared with the Cd treatment alone (Cd), the exogenous MT weakened the ROS levels under Cd stress. In the treatment of 50 μmol/L MT (Cd+50M), H_2_O_2_ and MDA were reduced by 65.4% and 45.4%, respectively (**Figures 2A, B**), while the content of O_2_^-^ was reduced by 63.6% after adding 100 μmol/L MT (Cd+100M) (**Figure 2C**). However, the ROS content under the 200 μmol/L MT (Cd+200M) treatment showed no significant change.

The activities of SOD and CAT in *B. striata* tubers were significantly higher under 250 μmol/L Cd treatment (Cd) than that of the control (CK), while the activities of POD and APX showed no significant change. Compared with the Cd treatment alone (Cd), the activities of SOD, POD, CAT and APX increased significantly after adding 50μmol/L MT (Cd+50M), After adding 100 μmol/L MT (Cd+100M), the activities of SOD and POD increased significantly, while the CAT activity decreased significantly, while the APX activity showed no obvious change. After adding 200 μmol/L MT (Cd+200M), the activities of POD and APX significantly increased, but the activities of SOD and CAT showed no significant difference (**Figures 2D-G**). The results preliminarily showed that 50 μmol/L MT was more effective than those of 100 μmol/L MT and 200 μmol/L MT in reducing the toxicity of Cd.

### 3.3 Cd concentration in *B. striata* tubers

The Cd concentration in *B. striata* tubers was assessed following the application of MT under Cd stress conditions (**Supplementary Figure S2**). Compared with the control group, Cd concentration exhibited a significant increase under treatment with 250 μmol/L Cd. Relative to the Cd-only treatment, the addition of MT led to a significant reduction in Cd accumulation. Although no significant difference was observed between the 50 μmol/L MT (Cd+50M) and 100 μmol/L MT (Cd+100M) treatments, both resulted in significantly lower Cd concentrations than the 200 μmol/L MT (Cd+200M) treatment.

### 3.4 Effects of MT on the nutritional quality of *B. striata*

**Figure 3:**
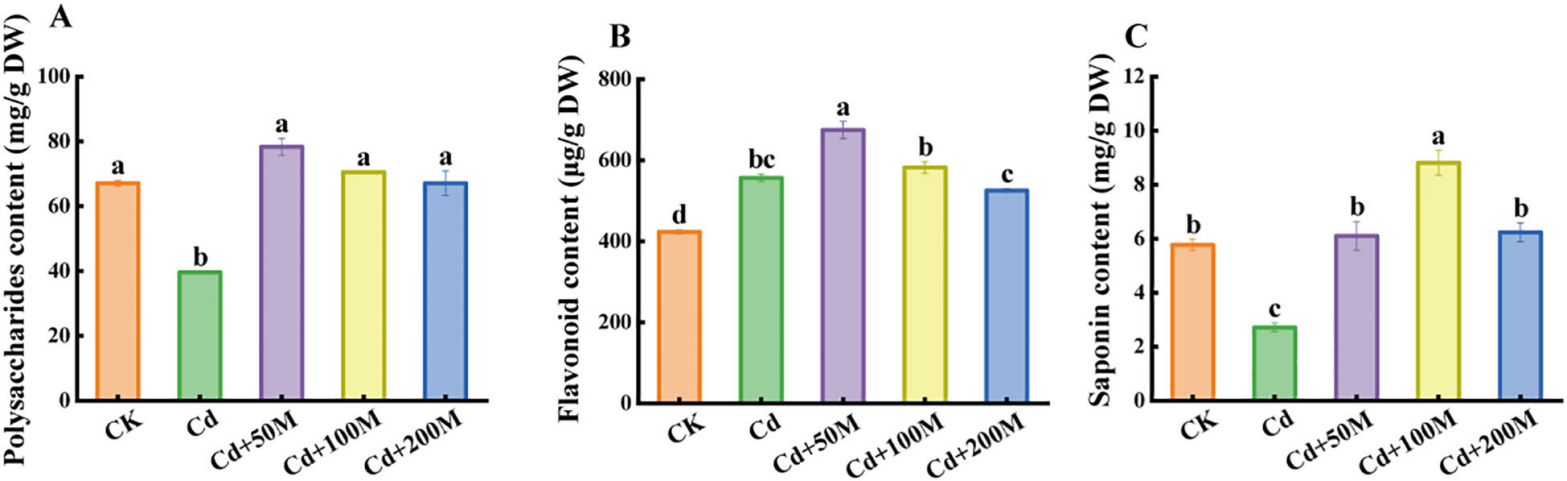
Effects of different MT treatments on the content of BSP **(A)**, flavonoids **(B)**, and saponins **(C)** in *B. striata* tubers under Cd stress. Letters (a, b, c) mean significant difference at p < 0.05.

To study the effect of MT treatment on the nutritional quality of *B. striata* under Cd stress, the quality traits of the tubers were investigated. The results indicated that the contents of polysaccharides and saponins in *B. striata* tubers under Cd-only treatment (Cd) were significantly lower than those in the control group (CK), while the flavonoid content increased significantly. Moreover, the contents of polysaccharides and saponins in groups treated with different MT concentrations were significantly higher than those in the group treated with 250 μmol/L Cd. Compared with the single Cd treatment (Cd), the 50 μmol/L MT treatment (Cd+50M) maximally increased the contents of polysaccharides and flavonoids by 98.0% and 4.6%, respectively (**Figures 3A, B**). Meanwhile, the saponin content reached the highest under the 100 μmol/L MT treatment (Cd+100M), which was 3.24 times that of the single Cd treatment (**Figure 3C**). In general, the 50 μmol/L MT (Cd+50M) treatment significantly improved the nutritional quality of *B. striata* tubers.

### 3.5 Differential metabolite analysis

**Figure 4:**
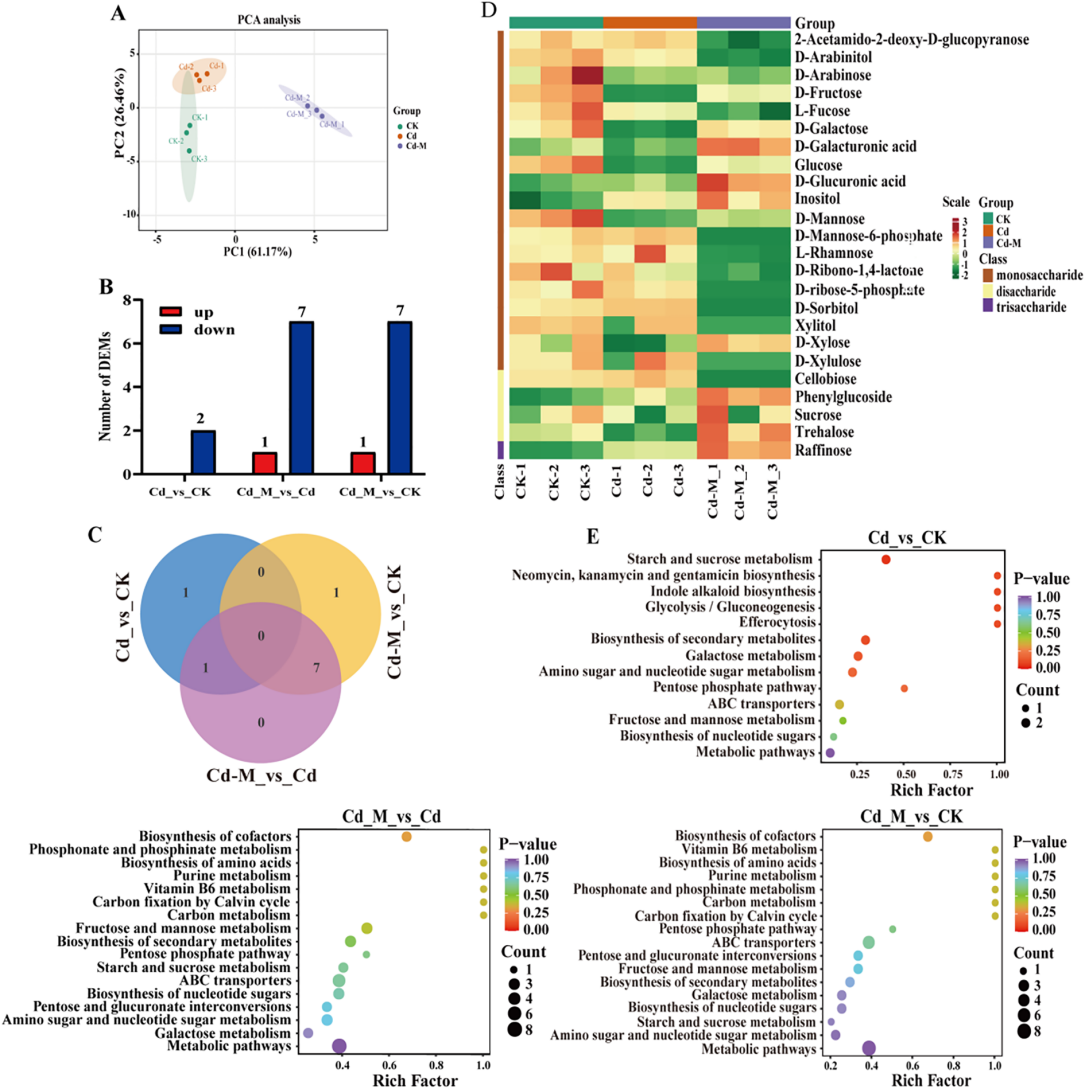
Metabolomics analysis of *B. striata* tube rs in different treatments. **(A)** The PCA analysis of DEMs amongCd_vs_CK, Cd_M_vs_Cd, Cd_M_vs_CK; **(B)** The number distribution of DEMs among Cd_vs_CK, Cd_M_vs_Cd, Cd_M_vs_CK; **(C)** Venn diagram of DEMs amongCd_vs_CK, Cd_M_vs_Cd, Cd_M_vs_CK; **(D)** Hierarchical clustering of DEMs among Cd_vs_CK, Cd_M_vs_Cd, Cd_M_vs_CK; **(E)** KEGG pathway enrichment map of DEMs among Cd_vs_CK, Cd_M_vs_Cd, Cd_M_vs_CK. CK refers to the control, Cd refers to 250 μmol/L Cd treatment, Cd_M refers to the treatment of 250 μmol/L Cd+50 μmol/L MT.

We employed a targeted database comprising 32 compounds associated with glycometabolism to fully understand the chemical basis underlying polysaccharide accumulation in *B. striata* tubers. Glycometabolism-related data were generated through gas chromatography-tandem mass spectrometry (GC-MS)-based metabolic profiling of tubers subjected to three different treatments (CK, Cd, and Cd+M). A total of 10 DEMs were identified in the 3 treatment groups, including 8 monosaccharide, 1 disaccharide and 1 trisaccharide (**Supplementary Table S1**). Then, principal component analysis (PCA) was conducted on the metabolites under the three treatments, indicating that the different treatments showed strong spatial separation due to substantial differences. Furthermore, the three replicates of each treatment clustered closely, indicating reliable data (**Figure 4A**). The number of down-regulated DEMs was 2 in Cd_vs_CK. The up-regulated DEMs were significantly lower than that of down-regulated DEMs in Cd_M vs Cd, Cd_M vs CK (**Figure 4B**).

It can be seen from the Venn diagram (**Figure 4C**) that 1 DEM of Cd_vs_CK and Cd_M_vs_Cd is D-fructose. D-fructose was down-regulated in Cd_vs_CK, while up-regulated in Cd_M_vs_Cd. And there were 7 DEMs in Cd_M_vs_Cd and Cd_M_vs_CK, including 2-Acetamido-2-deoxy-D-glucopyranose, Cellobiose, D-Mannose-6-phosphate, D-ribose-5-phosphate, D-Sorbitol, Xylitol, and D-Xylulose, all of which were down-regulated in Cd_M_vs_Cd and Cd_M_vs_CK. Therefore, these DEMs can be used as candidate metabolites in response to MT-regulated Cd stress.

Hierarchical cluster analysis of the metabolite sets showed significant differences among the samples from different treatments, indicating that Cd stress (Cd) and 50 μmol/L MT intervention (Cd-M) had a significant regulation on the accumulation pattern of sugar metabolites (**Figure 4D**). The analysis of KEGG enrichment was performed on the all treatment combinations, only the pathway of aminoacyl-tRNA biosynthesis was significantly enriched (P-value<0.05) by DEMs in CK vs T1 (**Figure 2C**). The analysis of KEGG enrichment revealed that DEMs was significantly enriched in the pathway of starch and sucrose metabolism (P <0.1) in Cd_vs_CK (**Figure 4E**). In Cd_M_vs_Cd and Cd_M_vs_CK, DEMs showed enrichment in the pathways of metabolic pathways and ABC transporters. These results suggest that MT may affect the accumulation of sugar metabolites in *B. striata* under Cd stress through a variety of metabolic pathways.

### 3.6 Differential gene transcriptome analysis

The transcriptome response of tubers was investigated by high-throughput sequencing, and a total of 55.66 Gb of clean data were obtained. The average numbers of clean reads acquired from CK, Cd, and Cd_M were 45,535,412, 41,448,637, and 38,720,799, respectively. In addition, the average GC contents were 45.93% for CK, 46.79% for Cd, and 46.79% for Cd_M. The average Q20 values of CK, Cd, and Cd_M were 99.27%, 99.24%, and 99.23%, respectively, and the Q30 values were 96.26%, 96.20%, and 96.19%, respectively, indicating that the quality of the transcriptome sequencing data was very high (**Supplementary Table S2**). The raw data were uploaded to NCBI (PRJNA1330263). Moreover, a mixed assembly strategy for all samples was adopted, and 265,581 transcripts and 79,329 unigenes were identified after assembly using Trinity software, with average lengths of 1156 bp and 347 bp, respectively.

**Figure 5:**
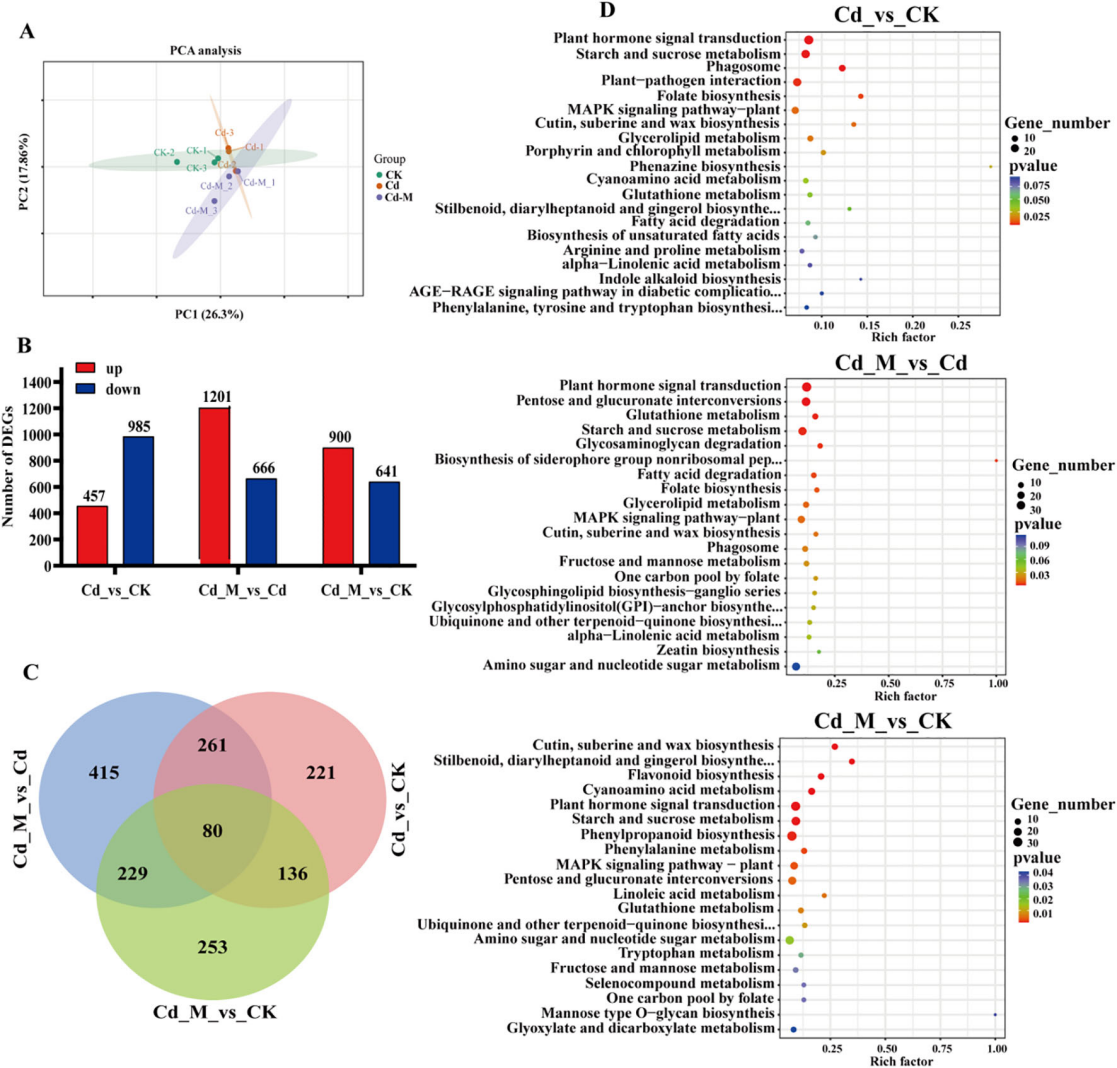
Transcriptome analysis of *B. striata* tubers in different treatments. **(A)** The PCA analysis of DEGs among Cd_vs_CK, Cd_M_vs_Cd, Cd_M_vs_CK; **(B)** The number distribution of DEGs among Cd_vs_CK, Cd_M_vs_Cd, Cd_M_vs_CK; **(C)** Venn diagram of DEGs amongCd_vs_CK, Cd_M_vs_Cd, Cd_M_vs_CK; **(D)** KEGG pathway enrichment map of DEGs among Cd_vs_CK, Cd_M_vs_Cd, Cd_M_vs_CK. CK refers to the control, Cd refers to 250 μmol/L Cd treatment, Cd_M refers to the treatment of 250 μmol/L Cd+50 μmol/L MT.

Principal component analysis of the gene expression levels of the 9 samples (**Figure 5A**) revealed that the differences between the two principal components were significant, accounting for 26.3% and 17.86% of the total variance, respectively. The biological replicates were closely clustered, indicating significant differences in the transcriptome results. There were 1442, 1867 and 1541 differentially expressed genes in the comparisons of Cd_vs_CK, Cd_M_vs_Cd and Cd_M_vs_CK, respectively, of which 457, 1201 and 900 were up-regulated and 985, 666 and 641 were down-regulated (**Figure 5B**). It indicated that the effects on the gene expression were significant in *B. striata* tubers under Cd treatment or the addition of MT. Venn analysis identified 80 genes co-expressed across all treatment comparisons (**Figure 5C**).

KEGG enrichment analysis revealed that the Cd vs CK, Cd_M vs Cd, and Cd_M vs CK groups contained 5, 7, and 10 DEGs with enriched metabolic pathways, respectively (p < 0.01). Interestingly, plant hormone signal transduction and starch and sucrose metabolism were observed in all groups (**Figure 5D**). Furthermore, DEGs in Cd vs CK were enriched in phagosomes, plant-pathogen interaction, and folate biosynthesis, while those in Cd_M vs Cd were enriched in pentose and glucuronate interconversions, glutathione metabolism, glycosaminoglycan degradation, biosynthesis of siderophore group nonribosomal peptides, and fatty acid degradation. This indicated that under Cd stress, MT activates the metabolism of carbohydrates and antioxidants in tubers.

### 3.7 Investigation of BSP synthesis-related gene and metabolites expression pattern

**Figure 6:**
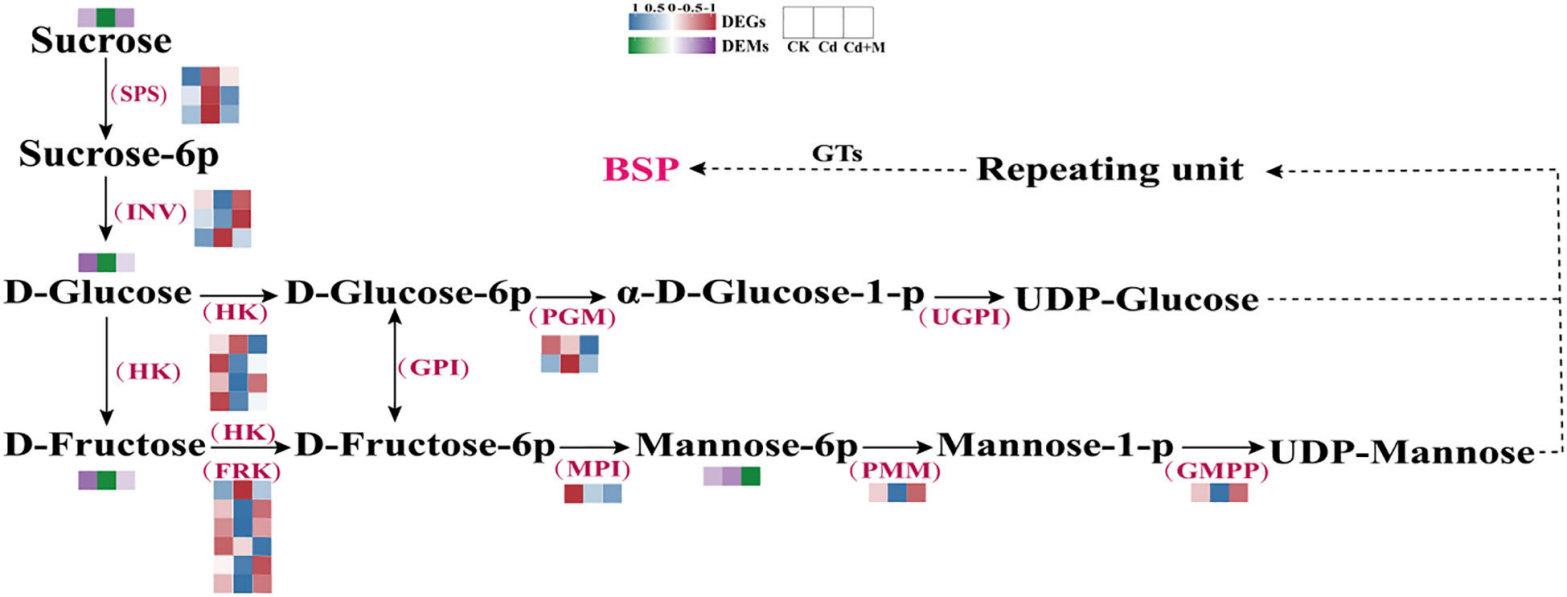
BSP biosynthesis pathway.

To investigate the mechanism by which MT regulates the accumulation of BSP under Cd stress, the expression patterns for DEGs and DEMs involved in BSP biosynthesis were examined (**Figure 6**). Here, 21 genes were identified that encode essential enzymes participating in the BSP biosynthesis pathways, including sucrose phosphate synthase (SPS), invertase (INV), hexokinase (HK), fruktokinase (FRK), phosphoglucomutase (PGM), mannose-6-phosphate isomerase (MPI), phosphomannomutase (PMM), and mannose-1-phosphate guanyltransferase (GMPP) (**Supplementary Table S3**). PMM and GMPP are crucial enzymes involved in the biosynthesis of BSP. The expression patterns of these genes exhibited significant differences under various treatments (**Figure 6**). For instance, the expression of *PMM* and *GMPP* genes was down-regulated under Cd treatment alone (Cd), while *SPS* gene expression was up-regulated. However, following combined treatment with 50 μmol/L MT treatment (Cd+M), *PMM* and *GMPP* genes showed up-regulated expression, whereas *SPS* and *PGM* genes were down-regulated. Cluster analysis of the heatmap revealed that among the six *FRK* genes, four exhibited significantly reduced expression levels under Cd stress conditions. Additionally, there are 4 sugar metabolites (**Supplementary Table S4**), including: Glucose, D-Fructose, D-Mannose-6-phosphate, Sucrose. It was observed that, the metabolites sucrose, Glucose, and D-Fructose showed a decrease under Cd treatment alone (Cd), while the metabolite D-Mannose-6-phosphate showed an increase. In contrast, the metabolite sucrose showed an increase, while the metabolite D-Mannose-6-phosphate showed a decrease 50 μmol/L melatonin treatment (Cd+M).

### 3.8 Correlation analysis between DEGs and DEMs

**Figure 7:**
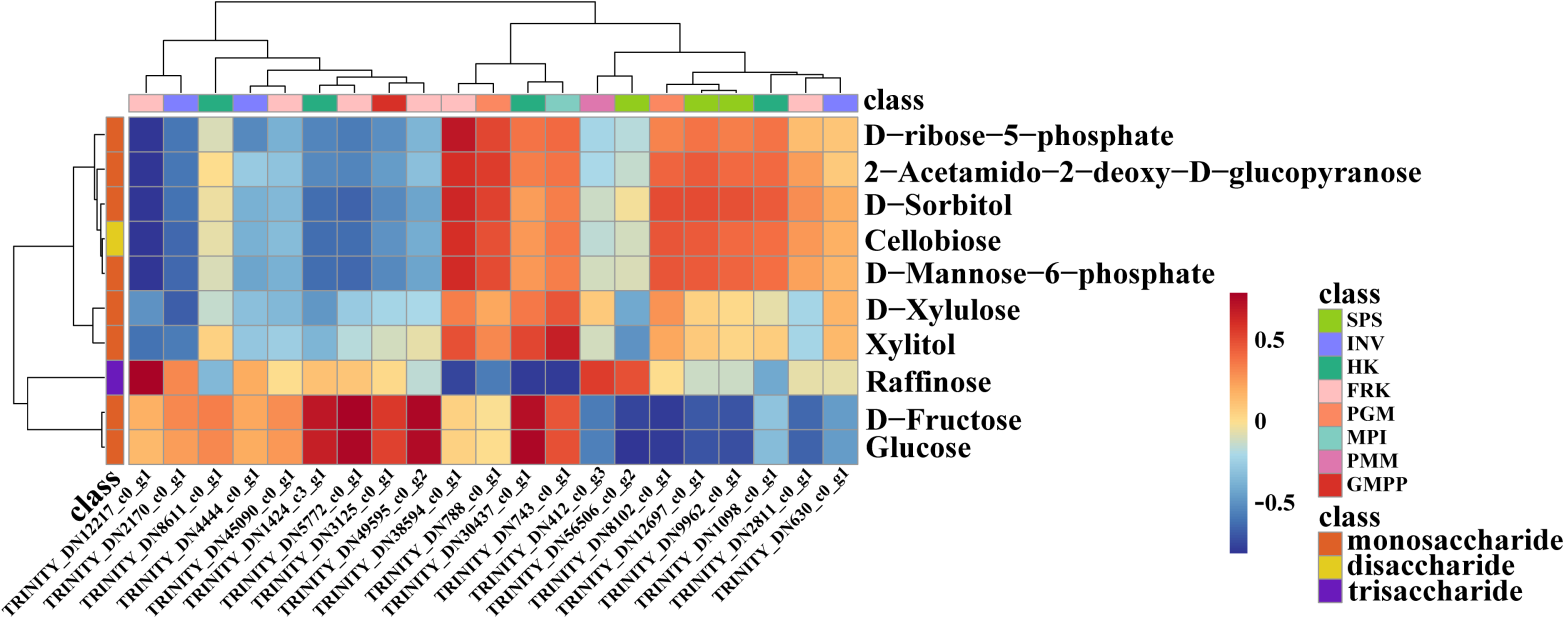
The correlation heatmap of DEGs and DEMs related to BSP synthesis.

To further explore the candidate genes that contributed to the accumulation of BSP content, the correlation analysis of the amount of DEMs and the expression of DEGs was performed (**Figure 7**). Through Pearson correlation analysis, a total of 10 structural genes were identified to be strongly correlated with these metabolites (|r| > 0.7), among which eight genes showed strong correlations with metabolites involved in the BSP metabolic pathway. This implied that these genes may be associated with BSP accumulation. For example, the correlation heatmap revealed that both *HK4* (TRINITY_DN30437_c0_g1), *FRK1*(TRINITY_DN5772_c0_g1) and *FRK6* (TRINITY_DN49595_c0_g2) were positively correlated with glucose and D-fructose. Additionally, *HK2* (TRINITY_DN1424_c3_g1) was also positively correlated with D-fructose (**Figure 7**).

**Figure 8:**
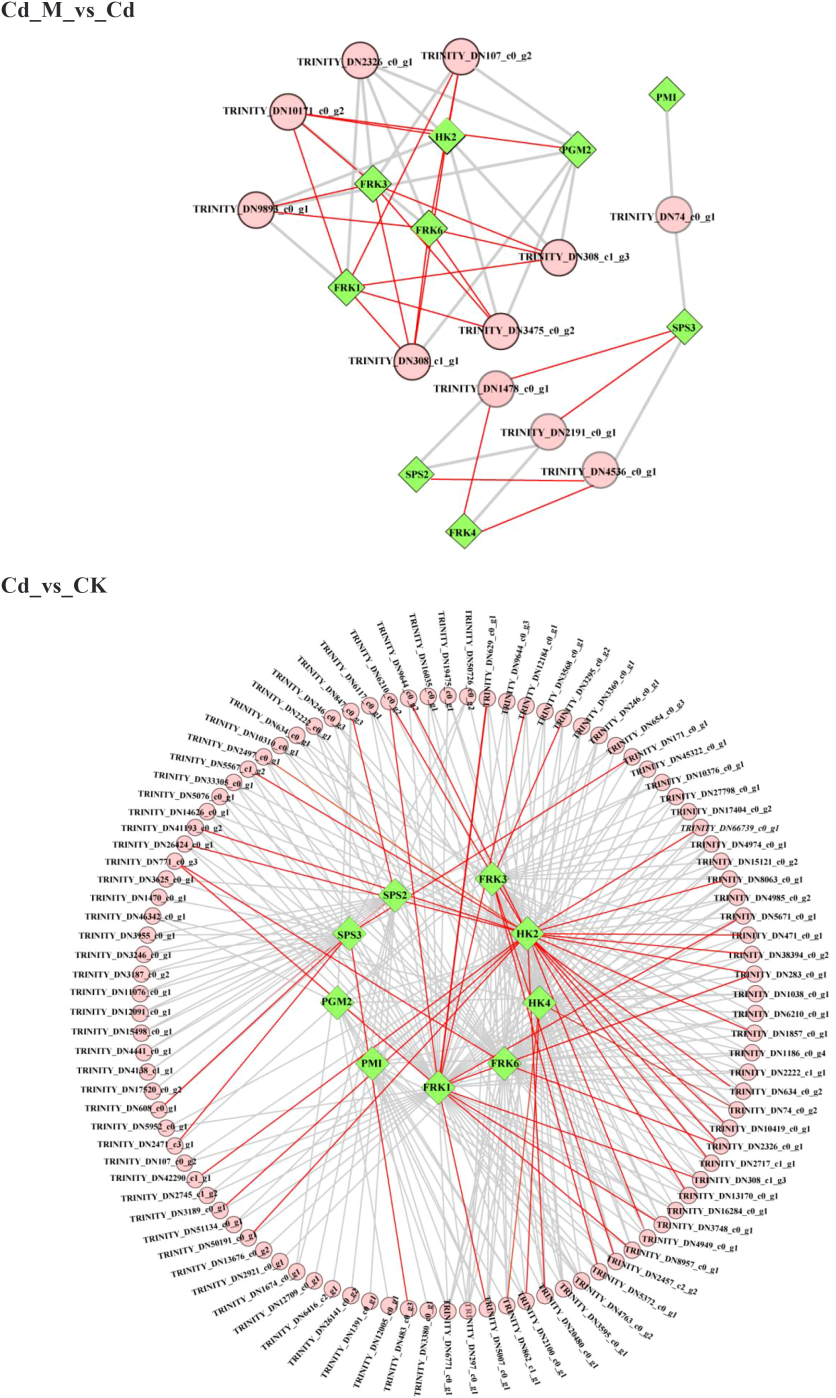
Coexpression network analysis of hub genes (green rhombus) and TFs (pink circles). The red line reflects a correlation coefficient between the two nodes larger than 0.95.

Meanwhile, to further investigate the potential transcriptional regulation of the BSP biosynthesis pathway, we constructed a TF-DEG correlation network (|r| > 0.8) based on the 10 screened DEGs (**Figure 8**). The results revealed that in the comparison between the Cd_M and Cd groups, the five most upregulated genes were *FRK1* (TRINITY_DN5772_c0_g1), *FRK3* (TRINITY_DN12217_c0_g1), *FRK6* (TRINITY_DN49595_c0_g2), *HK2* (TRINITY_DN1424_c3_g1), and *PGM2* (TRINITY_DN8102_c0_g1), which also served as key nodes regulated by numerous TFs, For instance, *FRK3* (TRINITY_DN12217_c0_g1) showed a positive correlation with *bHLH* (TRINITY_DN308_c1_g1) and *bZIP* (TRINITY_DN10171_c0_g2), with correlation coefficients greater than 0.95 (p < 0.01). In addition, *FRK4* (TRINITY_DN38594_c0_g1) was also positively correlated with *MYB_related* (TRINITY_DN1478_c0_g1), and the correlation coefficient was greater than 0.95 (p<0.01). In the comparison between the Cd and CK groups, the five most upregulated genes, along with several key node genes regulated by TFs, were *HK2* (TRINITY_DN1424_c3_g1), *HK4* (TRINITY_DN30437_c0_g1), *FRK1* (TRINITY_DN5772_c0_g1), *FRK3* (TRINITY_DN12217_c0_g1), and *FRK6* (TRINITY_DN49595_c0_g2); among these, *HK2* (TRINITY_DN1424_c3_g1) was positively correlated with *WRKY* (TRINITY_DN283_c0_g1) and *ERF* (TRINITY_DN9644_c0_g2), while *HK4* (TRINITY_DN30437_c0_g1) was positively correlated with *MYB* (TRINITY_DN6771_c0_g1), with correlation coefficients greater than 0.95 (p < 0.01). Notably, *HK3* (TRINITY_DN30437_c0_g1) was significantly upregulated under both Cd_M and CK conditions. These findings suggest that *FRK1*, *FRK3*, *FRK6* and *HK2* are important genes involved in the melatonin-mediated regulation of BSP biosynthesis under Cd stress in *B. striata*, while specific transcription factors (TFs) such as *bHLH*, *bZIP*, *MYB*, and *MYB_relate* also play crucial roles as key transcription factors.

### 3.9 Validation of the DEGs by qRT−PCR

**Figure 9:**
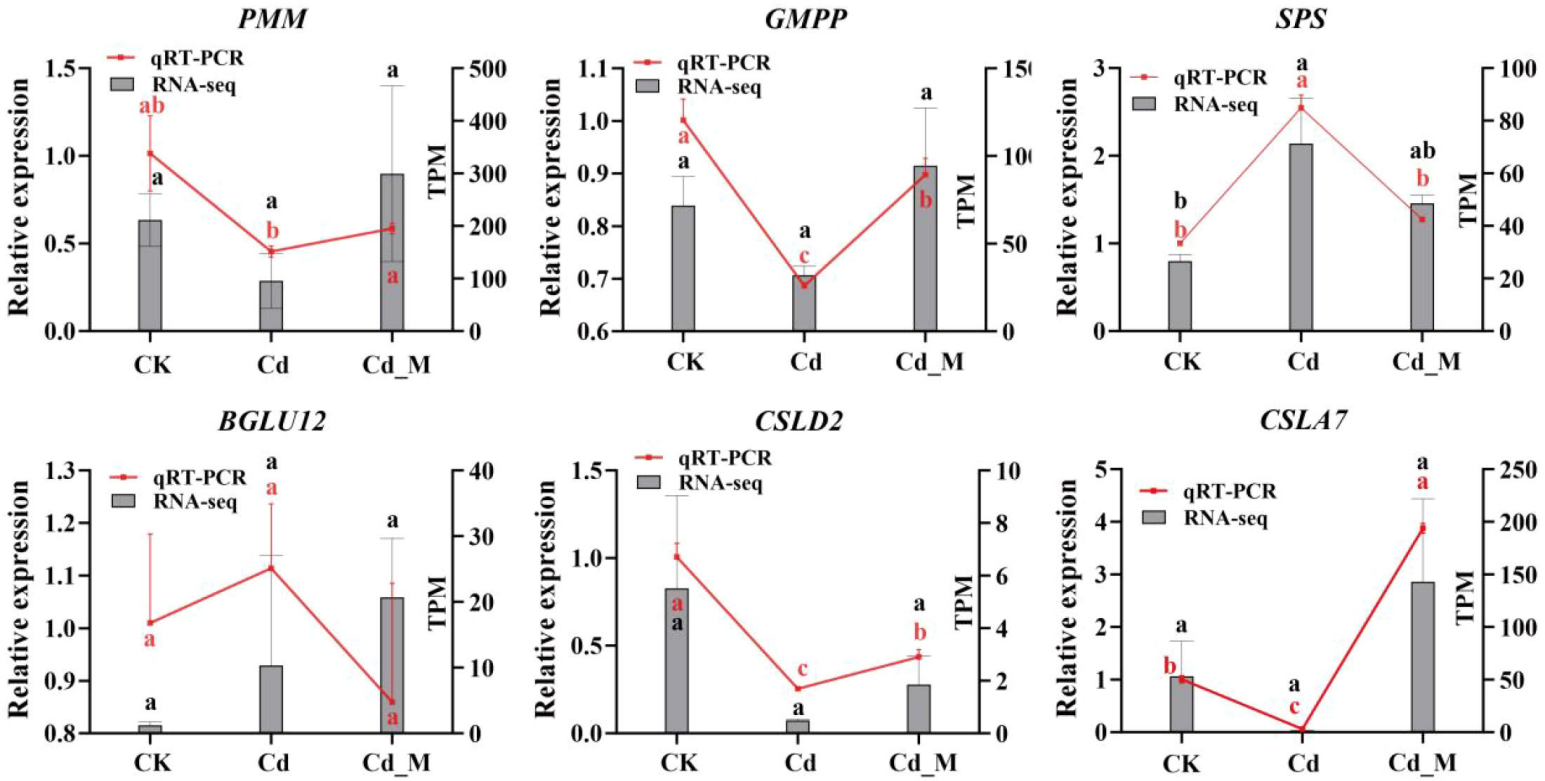
qRT-PCR validation of genes related to BSP biosynthesis of *B. striata* at three different treatments. *rRNA* gene was used as the reference gene. Letters (a, b, c) mean significant difference at p < 0.05.

To validate the transcriptome data, six genes associated with the BSP metabolic pathway were selected, and specific primers were designed based on the cDNA fragments of these genes (**Supplementary Table S5**). qRT-PCR was employed to analyze their expression levels in CK, Cd, and Cd_M groups. The relative expression levels of most candidate genes were consistent with the trends observed in the transcriptome data analysis, confirming the reliability and reproducibility of the RNA-seq results (**Figure 9**).

## 4 Discussion

### 4.1 Possible mechanisms by which MT alleviates Cd-induced growth inhibition and polysaccharide reduction in *B. striata*

Previous studies have demonstrated that MT functions as a growth regulator that promotes plant growth and development (Tan and Reiter, 2020). Moreover, it can re-establish redox homeostasis through enzymatic and non-enzymatic antioxidant defense systems, thereby alleviating Cd-induced oxidative stress (Gu et al., 2017; Kaya et al., 2019). In this study, the growth of *B. striata* seedlings treated with MT was superior to that of seedlings exposed to 250 μmol/L Cd stress alone (**Figure 1**, **Supplementary Figure S1**). This observation is consistent with previous studies demonstrating that MT application can enhance rapeseed growth under Cd stress (Menhas et al., 2022). Furthermore, under 250 μmol/L Cd stress, the activities of SOD and CAT as well as the content of ROS increased significantly, while the activities of POD and APX showed no significant changes. However, after the addition of 50 μmol/L MT, the activities of SOD, POD, CAT, and APX increased significantly, and the ROS content decreased significantly, indicating that MT may alleviate oxidative damage caused by Cd stress through activation of the reactive oxygen species-scavenging system, which consists of antioxidant enzymes, such as SOD, CAT, POD and APX (Ghuge et al., 2023). These findings are consistent with those reported in prior studies on tomato and *Brassica napus* (Hasan et al., 2015; Sami et al., 2020). Interestingly, our results demonstrated that with increasing concentrations of MT, the Cd concentration in *B. striata* tubers correspondingly increased. This phenomenon may be attributed to the concentration-dependent threshold effect of MT: when high concentrations of MT exceed the Cd defense threshold of plants, they promote Cd uptake and accumulation by reversing the regulation of transport genes, disrupting the chelation-compartmentalization system, and inducing oxidative stress. Previous studies have shown that MT can enhance the retention capacity of Cd in peanut (*Arachis hypogaea* L.) roots and reduce its translocation to seeds by upregulating the *AhNHL* gene, which promotes glutathione (GSH) metabolism and phenylpropanoid synthesis (Ren et al., 2025). Furthermore, the modulation of *IRT1*, *Nramp1*, *HMA2*, *HMA4*, and *HMA3* genes caused by melatonin could be responsible for intensifying Cd sequestration into the root vacuoles (Wang et al., 2019).

BSP, serving as a quality marker for assessing the quality of *B. striata*, exhibits multiple biological functions (Niu et al., 2020). In this study, the polysaccharide content exhibited a significant reduction under 250 μmol/L Cd stress alone, which is consistent with findings reported in studies on *Ganoderma lucidum* polysaccharides (Jiang et al., 2019). However, the polysaccharide content increased significantly following the addition of MT. Notably, the polysaccharide content in the 50 μmol/L MT treatment group was higher than that observed in the 100 μmol/L and 200 μmol/L MT treatment groups. This phenomenon can be attributed to the hormesis effect of MT on the regulation of plant secondary metabolism. At appropriately low concentrations, MT enhances polysaccharide accumulation by activating the polysaccharide biosynthesis pathway. Research indicates that MT modulates the expression of genes encoding key enzymes involved in glucose metabolism, including pectin lyase, UDP-glucose dehydrogenase, sucrose-phosphate synthase, hexokinase-1, and proteins associated with phosphorylation. This regulatory mechanism promotes the synthesis and interconversion of precursor molecules such as glucose and galactose, thereby ensuring an adequate supply of substrates for polysaccharide formation (Gu et al., 2024). In contrast, elevated concentrations of MT disrupt the carbon-nitrogen metabolic balance in plants. Given that tryptophan serves as a precursor for endogenous MT synthesis, excessive exogenous MT leads to competitive consumption of nitrogen resources. This results in reduced availability of carbon skeleton compounds, such as sucrose and hexose for polysaccharide biosynthesis, thereby indirectly inhibiting polysaccharide accumulation (Hassan et al., 2022). This suggests that appropriate MT treatment can significantly improve the quality of *B. striata* under Cd stress. Flavonoids and saponins are significant secondary metabolites that exhibit promising potential in the treatment of various diseases (Arab et al., 2013; He et al., 2021). In this study, the content of total saponins decreased significantly under Cd stress alone, and the content of flavonoids increased significantly. As antioxidants, flavonoids have both a 2,3-double bond and a 3-hydroxyl group in their basic structure, which can also enhance their antioxidant activity (Silva et al., 2002). When plants are subjected to abiotic stress, flavonoid accumulation is induced to reduce oxidative damage caused by ROS (Zheng et al., 2025). Following the addition of 50 μmol/L MT, the levels of total flavonoids and total saponins exhibited a significant increase. This observation is consistent with findings reported in previous studies on *Panax notoginseng* (Li et al., 2022). The research concluded that the application of MT enhanced the tolerance of *B. striata* to Cd stress. Furthermore, based on the systematically analysis of physiological and biochemical responses in *B. striata* seedlings under Cd exposure, it was determined that a concentration of 50 μmol/L MT exerted the most effective alleviation of Cd-induced toxicity.

### 4.2 Effects of MT on the expression of key enzyme genes involved in polysaccharide biosynthetic pathways

The biosynthesis of BSP is a complex process that primarily involves three sequential steps. First, guanosine diphosphate mannose (GDP-Mannose) and uridine diphosphate glucose (UDP-Glucose) are synthesized through the catalytic action of several key enzymes, including uridine diphosphate glucose pyrophosphorylase (UGP2), phosphoglucomutase (PGM), glucose-6-phosphate isomerase (GPI), MPI, PMM and GMP. Subsequently, these activated nucleotide sugars serve as donors for sugar residues in the formation of polysaccharide subunits. Finally, specific glycosyltransferases (GTs) mediate the polymerization of nucleoside diphosphate (NDP)-activated sugars into mature polysaccharides (Jiang et al., 2022). As an exogenous plant bioregulator, MT can modulate metabolite levels in plant polysaccharide metabolism by regulating the expression of associated genes and enhancing the activity of relevant enzymes, thereby improving plant tolerance to Cd stress (Gu et al., 2024).

HK and FRK play important roles in the biosynthesis of BSP. As a “gatekeeper enzyme” in sugar metabolism, HK catalyzes the phosphorylation of glucose or fructose in the polysaccharide biosynthesis pathway to generate glucose-6-phosphate (G6P) or fructose-6-phosphate (F6P), thereby providing essential precursors for the subsequent biosynthesis of polysaccharides (e.g., glycogen and glucomannan) (Hay, 2016). FRK uses ATP as the phosphate donor to phosphorylate free fructose into F6P or fructose-1-phosphate (F1P), which is the initial step in the production of plant polysaccharides (Damari-Weissler et al., 2006). In the present study, expression profiling of genes associated with BSP biosynthesis and corresponding metabolites revealed that under Cd stress, the transcript levels of 3 *HK* genes and 4 *FRK* genes were significantly downregulated. Following MT application, the expression levels of 4 *FRK* genes were upregulated, whereas the concentrations of glucose and D-fructose showed no significant changes (**Figure 6**). These findings are in alignment with the results reported in the previously studies on *Medicago sativa* (Gu et al., 2024) and *Vicia sativa* (Rui et al., 2016). Furthermore, PMM and GMPP are key enzymes involved in BSP biosynthesis. These two enzymes function synergistically to catalyze the formation of GDP-mannose, a critical precursor for BSP synthesis. Previous studies have demonstrated that GDP-mannose levels in plants increase significantly under stress conditions (He et al., 2017). Our current study demonstrated that exogenous MT application under Cd stress significantly upregulated the gene expression levels of *PMM* and *GMPP*, while markedly reducing the content of mannose-6-phosphate (**Figure 6**). This indicates that the application of MT may enhance the conversion rate of mannose-6-phosphate to GDP-mannose, thereby contributing to an increase in BSP content. Correlation analysis between DEMs and DEGs revealed that *HK4*, *FRK1*, and *FRK6* exhibited significant positive correlations with the levels of D-glucose and D-fructose. Furthermore, *HK2* showed a significant positive correlation with D-fructose (**Figure 7**). Our results indicate that the expression of these four genes is closely associated with alterations of saccharide metabolic levels in *B. striata* regulated by MT under Cd stress, suggesting their potential role as core regulatory factors through which MT enhances saccharide metabolism in *B. striata* under Cd stress.

### 4.3 Potential regulation of transcription factors by MT in mediating polysaccharide metabolism under Cd stress

In plant polysaccharide synthesis, TFs bind to promoter regions of biosynthetic genes in a sequence-specific manner, thereby modulating their expression. In this study, by the co-expression network analysis, TFs such as *MYB*, *MYB-related*, *bHLH*, and *bZIP* were found to be involved in regulating BSP biosynthesis. Notably, the transcriptional levels of these TFs showed strong correlations with the expression of key enzyme genes *FRK1*, *FRK3*, *FRK6*, and *HK2*. And compared with Cd treatment, the expression levels of most of the TFs were upregulated under the 50 μmol/L MT (Cd+50M) treatment. Within the TF family, *MYB* represents one of the largest and most multifunctional families in plants. Its members are classified into four major categories-*1R-MYB*, *2R-MYB*, *3R-MYB*, and *4R-MYB*-based on the number and arrangement of *MYB* repeat domains. These members play extensive roles during plant growth and development (Song, 2025). *MYB46* enhances the expression of *AtGELP7* through downstream regulatory factors in *Arabidopsis*, thereby influencing cell wall polysaccharide acetylation and sugar distribution (Rastogi et al., 2025). And overexpression of *DoMYB75* in *A. thaliana* can increase the content of water-soluble polysaccharides (WSP) in seeds by approximately 14% (He et al., 2019). Liu et al. found that the *TaMYB44* gene can directly bind to the promoters of starch synthesis-related genes, including *Sus1-A1*, *LDB1*, and *TPP-D1*, and repress their expression. Moreover, the study revealed that T*aWDR1* interacts with *TaMYB44* and counteracts its transcriptional repression activity, thereby enhancing starch synthesis in wheat (Liu et al., 2025). Our results demonstrated that the expression level of *MYB* (TRINITY_DN107_c0_g2) was positively correlated with those of *FRK1* and *HK2* (r > 0.95). Furthermore, under Cd stress, *MYB* (TRINITY_DN107_c0_g2) exhibited relatively low expression. However, its expression was significantly upregulated following MT treatment. This suggested that this gene may serve as a potential regulatory factor in BSP synthesis pathway in response to MT-mediated alleviation of Cd stress. Furthermore, the *bHLH* family represents one of the most extensive families of transcription factors and plays critical roles in a wide range of biological processes. For example, the *bHLH* transcription factor *PcbHLH*, identified in Polygonatum cyrtonema, exhibits strong protein-protein interactions with five key enzymes involved in the polysaccharide biosynthetic pathway. This indicates a potential regulatory function of *PcbHLH* in polysaccharide biosynthesis through the modulation of these enzymatic activities (Wang Z.W. et al., 2025). In this study, *bHLH* (TRINITY_DN308_c1_g1) was found to be strongly positively correlated with *FRK1* expression (r > 0.95). Similarly, the expression level of *bHLH* (TRINITY_DN308_c1_g1) was relatively low under Cd stress, whereas it was significantly upregulated following MT treatment. Although *MYB* and *bHLH* tfs perform distinct functions in polysaccharide biosynthesis across plant species, it has been established that both TF families contribute to the regulation of this metabolic pathway. Furthermore, significant correlations were observed between *bZIP* expression and *FRK1*, as well as between *MYB-related* and *FRK4*. Previous studies have suggested that *bZIP* may regulate genes involved in ginseng polysaccharide biosynthesis (Fang et al., 2022). *MYB-related* tfs participate in diverse biological processes in plants. Overexpression of the wheat *MYB-related* tf TaMYB13–1 has been shown to upregulate all three classes of fructosyltransferase genes, ultimately enhancing fructan accumulation (Kaya et al., 2019). These results demonstrate that the coordinated regulation of TFs and DEGs plays an essential role in mediating the response of BSP biosynthesis to MT under Cd stress. The identification of these TFs provides a theoretical foundation for further elucidating the regulatory mechanisms through which MT influences the BSP biosynthetic pathway.

## 5 Conclusion

The results indicate that MT treatment (50 μmol/L) alleviates oxidative damage by enhancing the activity of the antioxidant defense system, increasing the levels of osmoregulatory substances, polysaccharides, and total flavonoids, while reducing ROS levels. This ultimately promotes the growth and development of *B. striata* under Cd contamination. Integrated transcriptomic and metabolomic analyses revealed the potential regulatory mechanism of MT on BSP biosynthesis in tubers under Cd stress, identifying *FRK1, FRK3, FRK6* and *HK2* as key genes involved in BSP synthesis. Furthermore, the coexpression network analysis showed that transcription factors such as *MYB*, *MYB_related*, *bHLH* and *bZIP* played important roles in the biosynthesis of BSP. MT treatment primarily promotes the upregulation of genes related to BSP synthesis by modulating the expression of these transcription factors, thereby increasing total polysaccharide content. This research contributes to understanding the regulatory network of MT on BSP biosynthesis under Cd stress and the role of MT in mitigating Cd-induced oxidative damage, providing important insights for exploring MT-mediated regulation of plant growth under heavy metal stress.

## Data Availability

The data presented in the study are deposited in the NCBI repository, accession number PRJNA1330263.
