# Supplementary material for: Melatonin mitigates cadmium-induced oxidative damage and modulates polysaccharide biosynthesis in Bletilla striata
Source: Front Plant Sci. 2025 Nov 26;16:1713721. doi: 10.3389/fpls.2025.1713721 (PMC12689973; doi:10.3389/fpls.2025.1713721)
Supplement: Supplementary file 1 [file DataSheet1.docx]

**Fig S1.** Phenotypic of *Bletilla striata* seedlings leave under Cd stress and adding melatonin.


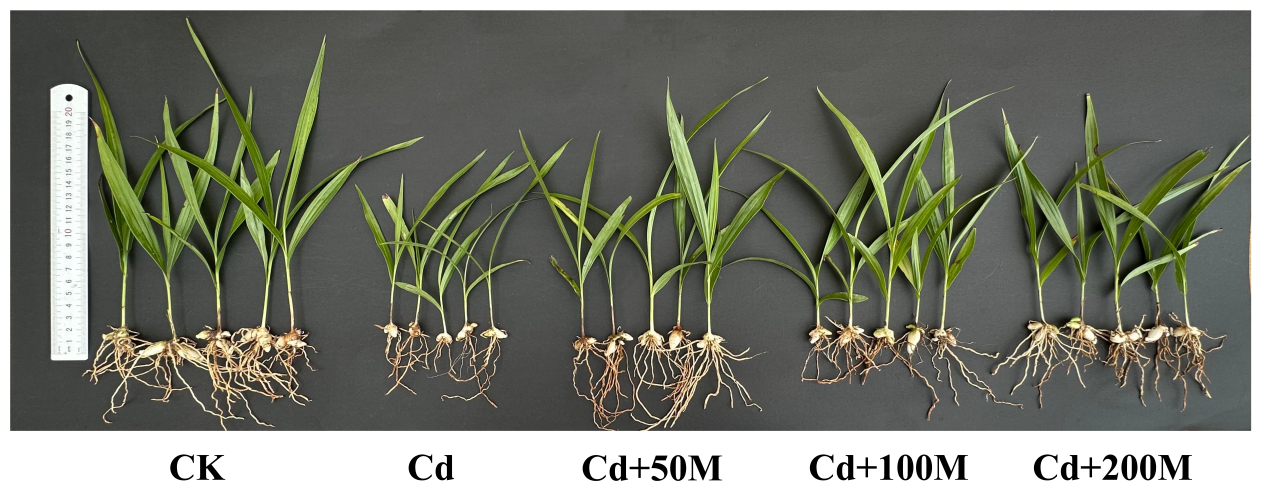


Note: CK, the control treatment; Cd, 250 µmol/L Cd treatment; Cd+50M, the treatment of 250 µmol/L Cd+50 µmol/L MT; Cd+100M, the treatment of 250 µmol/L Cd+50 µmol/L MT; Cd+100M, the treatment of 250 µmol/L Cd+50 µmol/L MT


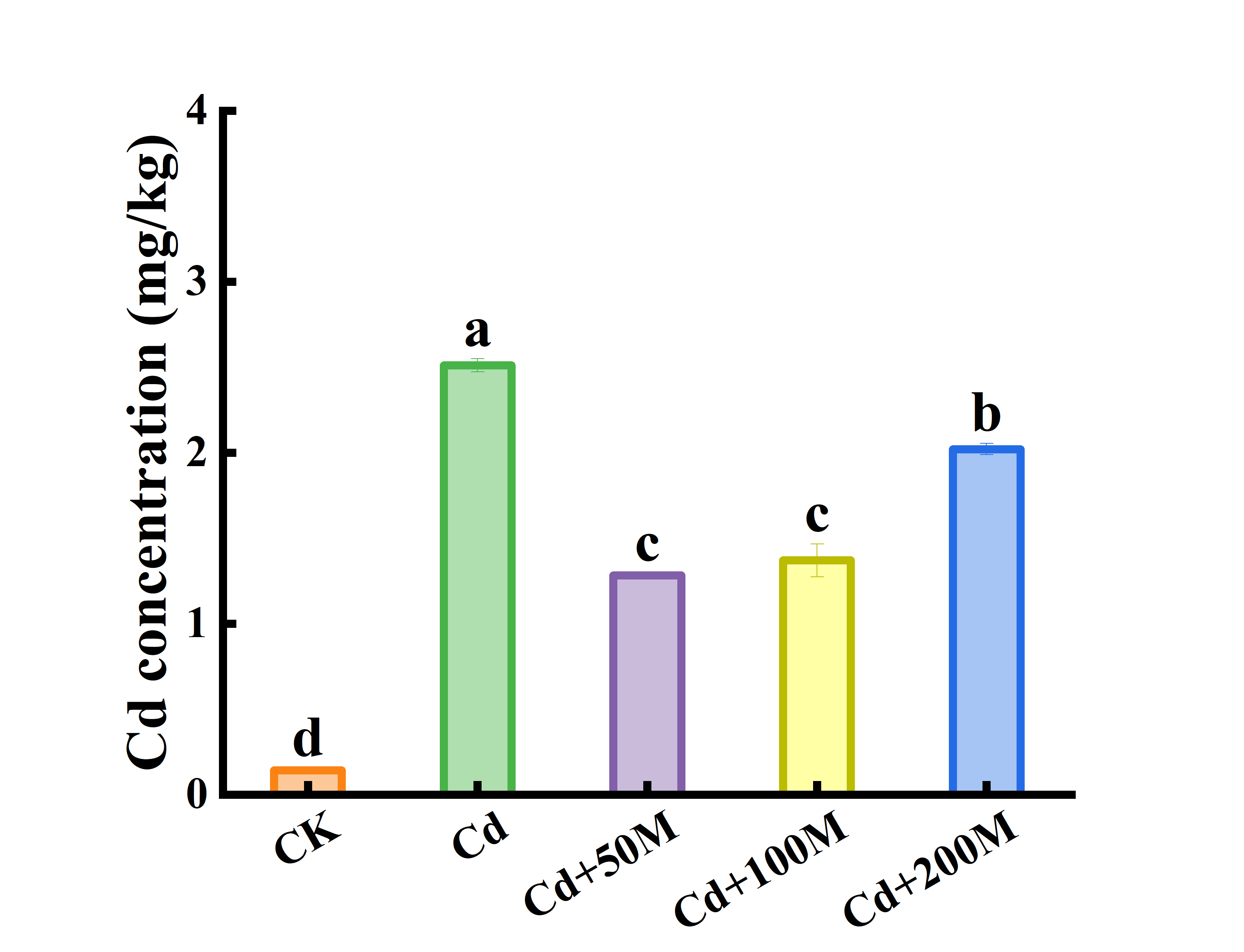
**Fig S2.** Cd concentration in *Bletilla striata* tubers leave under Cd stress and adding melatonin.

Note: CK, the control treatment; Cd, 250 µmol/L Cd treatment; Cd+50M, the treatment of 250 µmol/L Cd+50 µmol/L MT; Cd+100M, the treatment of 250 µmol/L Cd+50 µmol/L MT; Cd+100M, the treatment of 250 µmol/L Cd+50 µmol/L MT. Letters (a, b, c, d) mean significant difference at p < 0.05.
